# Randomized controlled trial of computerized cognitive behavioural therapy for depressive symptoms: effectiveness and costs of a workplace intervention

**DOI:** 10.1017/S0033291713001323

**Published:** 2013-06-24

**Authors:** R. Phillips, J. Schneider, I. Molosankwe, M. Leese, P. Sarrami Foroushani, P. Grime, P. McCrone, R. Morriss, G. Thornicroft

**Affiliations:** 1 Institute of Psychiatry, King's College London, London, UK; 2 Institute of Mental Health, University of Nottingham, Nottingham, UK; 3 University of New South Wales, Sydney, NSW, Australia; 4 Cambridge University Hospital NHS Foundation Trust, Cambridge, UK

**Keywords:** CBT, depression, stress, workplace

## Abstract

**Background:**

Depression and anxiety are major causes of absence from work and underperformance in the workplace. Cognitive behavioural therapy (CBT) can be effective in treating such problems and online versions offer many practical advantages. The aim of the study was to investigate the effectiveness of a computerized CBT intervention (MoodGYM) in a workplace context.

**Method:**

The study was a phase III two-arm, parallel randomized controlled trial whose main outcome was total score on the Work and Social Adjustment Scale (WSAS). Depression, anxiety, psychological functioning, costs and acceptability of the online process were also measured. Most data were collected online for 637 participants at baseline, 359 at 6 weeks marking the end of the intervention and 251 participants at 12 weeks post-baseline.

**Results:**

In both experimental and control groups depression scores improved over 6 weeks but attrition was high. There was no evidence for a difference in the average treatment effect of MoodGYM on the WSAS, nor for a difference in any of the secondary outcomes.

**Conclusions:**

This study found no evidence that MoodGYM was superior to informational websites in terms of psychological outcomes or service use, although improvement to subthreshold levels of depression was seen in nearly half the patients in both groups.

## Introduction

Depression and anxiety are recognized as major causes of work underperformance and absence (Sanderson *et al.* 2007). Up to 9% of the UK population is likely to be affected by these treatable mental health issues (Singleton *et al.* 2001) that make an impact directly on the productivity of the labour force. Yet a survey of employers in 2009 found that only 22% had mental health policies in place, and concluded that ‘people with mental health disorders are unlikely to avoid prejudices in the workplace’ (Trajectory, 2010). This could discourage appropriate help-seeking.

Computerized interventions can be provided consistently to large numbers of people and appear more acceptable to individuals who regard formal mental health services as stigmatizing. They are less costly than face-to-face therapy and easy to access thanks to the growing use of the Internet. Two computerized cognitive behavioural therapy (cCBT) packages received approval from the National Institute for Clinical Excellence (NICE, 2006): one for panic and phobia (Fearfighter) and one for mild and moderate depression (Beating the Blues). Primary care providers in England and Wales were then instructed by NICE to make cCBT available to all patients (Department of Health, 2007).

Evidence about these and other cCBT packages remains promising but inconclusive, making it desirable to investigate the costs and benefits of each package in relation to specific diagnostic groups (Sarrami *et al.* 2011). With the exception of one small trial in the National Health Service (NHS) of Beating the Blues (Grime, 2004), at the outset of the present trial we could find no published studies of cCBT in workplace settings. That study found that Beating the Blues was beneficial for depression and anxiety at 1 month, but not at 3 and 6 months post-treatment. However, there are considerable differences between the earlier study and the present one: participants in Grime's study (Grime, 2004) accessed the programme via a personal computer in a private room in their occupational health department, a relatively controlled context, while in the present study a different programme (MoodGYM) was tested, accessed via the Internet from any location.

### Aim

The aim of the study was, through a pragmatic trial in the workplace, to measure the impact of an interactive cCBT programme (MoodGYM) on employees' work-related performance and psychological well-being, compared with that of an ‘attentional’ control (five websites with general information about mental health). The main hypothesis was that users of MoodGYM would experience less functional impairment at work than the control group.

## Method

### Design and data collection

The study was a two-arm, parallel randomized controlled trial with a 5-week intervention period and follow-up at 6 and 12 weeks. The trial (no. ISRCTN24529487; http://www.controlled-trials.com/) was designed to be administered mainly online. The MoodGYM developers were commissioned by the study investigators to construct a research portal that could allocate login identities, screen, take consent, randomize and direct participants to the appropriate arm of the study. This issued emails that prompted participants to complete assessments at 6 and 12 weeks. The trial manager could be contacted by email in case of problems, such as forgotten passwords. The portal was piloted in June–September 2009, and following revisions to the website the trial was launched in November 2009. The trial portal was closed to new recruits on 31 May 2011.

Occupational health sections of three large employers agreed to participate in the trial, by directing their staff to the website and promoting the opportunity internally. The workplaces were thus a convenience sample, which spanned, as it turned out, the transport, health and communications sectors. Employees were given confidential access to the trial website. Online screening of potential participants offered the option of joining the trial if they were aged over 18 years and met the following criterion: on the Patient Health Questionnaire-9 (PHQ-9; Kroenke & Spitzer, 2002) the employee scored 2 or more on five of the nine items, including 2 or more on item 1 (little interest in doing things) or item 2 (feeling hopeless). To be eligible the employee also had to confirm that at least one of the items identified as a problem for them made it difficult to work, take care of things at home, or get along with other people.

All participants were required to give a telephone number as a condition of joining the study. Weekly telephone calls were made, lasting about 10 min on average, with three purposes: to maintain engagement with the study; to screen for risk; and to collect service use data for costing purposes. The telephone input was provided to both arms of the trial by the Mental Health Research Network's clinical studies officers, who made up to five calls before recording a non-response.

### Interventions

MoodGYM is a freely available course developed at Australia National University (ANU) which allows participants to proceed at their own pace over five, 1 h-long modules, usually taken weekly (ANU, 2012). The websites selected for the control group were judged from a previous review of self-help in mental health to be reliable sources of information about mental health problems, and are listed in Table 1.

**Table 1.** Website links sent weekly to participants in the control arm

Website address	Title on trial portal
http://www.hse.gov.uk/stress	Stress at work
http://www.nhs.uk/Conditions/Mental-health/Pages/Introduction.aspx?url=Pages/What-is-it.aspx	NHS Choices: mental health
http://www.mentalhealth.org.uk/information/mental-health-a-z/	A to Z of mental health problems
http://www.bbc.co.uk/health/conditions/mental_health/emotion_stress.shtml	Tackling stress
http://www.rethink.org/living_with_mental_illness/rights_and_laws/index.html	Your rights to care and treatment

NHS, National Health Service.

### Risk of adverse events

The telephone interviewers also screened for risk of self-harm or suicide, and followed a protocol which permitted them to breach confidentiality if participants seemed so unwell that immediate professional care was indicated but they were unwilling or unable to access this without assistance. This was invoked twice. In addition, participants were told at the outset and reminded periodically in the telephone interviews that they should consult their general practitioner (GP) or occupational health department (as appropriate for each organization) if they had intentions to harm themselves. On six occasions telephone interviewers contacted the relevant emergency contact with the client's permission.

### Ethical oversight

A favourable ethical opinion was granted by Derbyshire Local Research Ethics Committee on 6 January 2009. The Mental Health Research Network adopted the study in February 2009. ANU's ethics committee also approved the study before the portal was piloted.

### Sample size

The power calculation was designed to detect a mean difference of 3 points on the Work and Social Adjustment Scale (WSAS; Mundt *et al.* 2002). This 3-point difference is judged to be clinically significant on the basis of the findings from Proudfoot *et al.* (2004) where outcomes for intervention participants were consistently at least 3 points lower on the WSAS than for the treatment-as-usual participants. With an assumed standard deviation of 9 (rounded up from 8.4 in the same study) and 80% power at a 5% significance level, we required 142 participants per arm to complete the study. Assuming a drop-out of 20%, a total of 355 participants was required. Based on estimates of withdrawal, refusal and loss to the study on a previous large trial of MoodGYM (Mackinnon *et al.* 2008) it was anticipated that 60% of those eligible to participate would be retained over 6 months. To achieve a final sample of 355, recruitment of 592 participants was anticipated.

### Randomization and blinding

A list was produced by the Nottingham Clinical Trials Unit to allow simple (unrestricted) randomization (Schulz & Grimes, 2002). Once potential participants had completed the screening questions, if eligible for inclusion in the trial, they were given a study ID, allocated through the website, and they were then invited to join the trial. If participants consented, they were randomized by the portal designers at ANU. In this way the randomization status of participants was concealed from their employers and from the research team until the study was completed.

The study design aimed to be double-blind. Participants were told that they were participating in a trial of ‘online self-help’, comparing two approaches, and researcher bias was avoided completely because most data for the outcome measures were collected online. However, the telephone interviewers who recorded service use measures for economic analysis were not blind to the randomization status of the participants.

### Measures

#### Outcomes

The WSAS (Mundt *et al.* 2002) was used to measure the primary outcome: subjective, work-related performance. Besides having face validity in relation to employers' concerns, given the workplace focus of this trial, the WSAS is a brief, reliable and valid test, which has high correlations between clinician and self-report versions (Mundt *et al.* 2002). These considerations made the WSAS suitable for this study, in which online data collection and self-reporting were intended. The WSAS scale ranges from 0 to 40, with higher scores indicating more disability. It rates responses 0 (‘not at all’) to 8 (‘very severely’), and states: ‘People's problems sometimes affect their ability to do certain day-to-day tasks in their lives. To rate your problems look at each section and determine on the scale provided how much your problem impairs your ability to carry out the activity.’ The five items are work, home management, social leisure activities, private leisure activities, and family and relationships.

Secondary outcomes rated were: depression, using the PHQ-9 (Kroenke & Spitzer, 2002); Clinical Outcomes in Routine Evaluation (CORE10; Evans *et al.* 2002); and generalized anxiety disorder (GAD; Spitzer *et al.* 2006). Advantages of the PHQ-9 include its brevity and its construct and criterion validity. In addition to measuring diagnostic thresholds, the PHQ-9 can assist in estimating the severity of depressive disorders. Scores on the PHQ-9 range from 0 to 27, with higher scores indicating more depressive symptoms.

The CORE10 score can range from 0 to 40, with higher scores indicating that individuals are reporting more problems and experiencing more distress. The GAD score can range from 0 to 21, with higher scores indicating greater anxiety. Health-related quality of life was measured with the five-domain EuroQol (EQ-5D; Brooks *et al.* 1991). Each of the five domains (mobility, self-care, usual activities, pain/discomfort, anxiety/depression) is rated as 1 (no problem), 2 (moderate problems) or 3 (major problems).

Other measures included were self-assessed absence from work and acceptability of the online process. Service use [Client Service Receipt Inventory (CSRI); Beecham & Knapp, 2001] and quality of life (EQ-5D) were measured to permit cost-effectiveness analysis, reported here.

#### Demographic information

All those individuals who consented to participate provided basic demographic data online: gender, age, marital status, alcohol consumption, education and details of their employment.

#### Service use and sick leave

Telephone interviewers recorded the use of health and social care services by study participants with an adapted version of the CSRI, incorporating additional questions regarding lost employment: ‘In the past six months/week have you had any days off due to ill-health?’ and ‘If so, how many of these days would you say were due to your mental health?’

### Statistical analysis

#### Sample profile

The characteristics of the trial participants were compared with the whole workforce for each organization involved in the trial. The demographics of participants who completed screening and chose to proceed were compared with those who completed screening and did not proceed, and those who completed follow-ups were compared with non-completers. The sensitivity of the results of the main analysis to possible bias deriving from gender imbalance and drop-out was explored. We also investigated variation in participants' engagement with MoodGYM.

#### Loss to follow-up and other missing data

Where no more than 20% of items were missing, baseline items were imputed using the mean response to the valid questionnaire items. The WSAS data were complete at baseline so no imputation was performed. For the CORE10, PHQ-9 and GAD we imputed the total score based on the mean score of the valid responses; if less than 80% items were answered then the total was left missing. No imputation was conducted on outcome scores.

#### Main analysis

Data were analysed using Stata 11.2 (Timberlake Consultants, UK) according to the intention-to-treat principle; patients' data were analysed in the treatment groups to which they were randomized irrespective of treatment received as long as outcome data were available, including imputed baseline data as determined above.

The main research question was addressed by a single statistical model to estimate the difference in mean outcome (WSAS) of scores between participants randomized to MoodGYM and control across the two follow-up points (6 and 12 weeks). A double-sided 5% significance level was used for this analysis. No multiple testing adjustments were made to the significance level for the other analyses, which are regarded as exploratory.

A linear, mixed-effect model for longitudinal data (random intercept model) was used to estimate, using maximum likelihood, the difference between treatment arms in WSAS score at 6 and 12 weeks overall (taking account of any time trends). This approach allowed the simultaneous modelling of the two follow-up time points, thus reflecting the estimated difference in randomized groups across the entire follow-up period. This permitted us to use the data from follow-up assessments at both time points and to take account of the impact of time on the participants' outcomes.

The assumption of normality for the residuals was checked visually from probability plots. All participants with outcome data available were included in all models, whether they completed both 6- and 12-week follow-ups, or only one.

The pre-specified covariates that were included in the model consisted of the baseline outcome score, the randomization group and the organization. Time was included to estimate the time trends over the whole sample. An interaction between time and intervention was also tested for evidence of a differential effect over time.

Baseline variables that were associated with missing outcomes were investigated so that they could be included in models and minimize bias due to non-response.

#### Exception to the protocol

Due to a technical error in the research portal, a number of individuals who had more severe mental health problems were not excluded as the protocol intended. A total of 101 individuals who consented to participate were subsequently found to have brain injury or stroke or to be receiving CBT or treatment for bipolar disorder. Instead of being excluded by the portal, 67 went on to register as part of the trial, and 41 continued to complete the baseline measures and were therefore randomized (22 to MoodGYM and 19 to the control arm), 23 completed the 6-week follow-up quiz and 20 completed the 12-week follow-up. When this error came to light it was the subject of considerable discussion; the research team's decision was to retain in the sample the individuals who gave their data in good faith, that this was consistent with the trial's pragmatic design and the most ethical way to proceed. We fully explored the impact of this decision on the results and found that, while the mean WSAS score was higher for this subgroup of participants with more severe mental health problems, there were no differences on the other baseline variables and their inclusion in the final analyses made no difference to the results of the analyses.

#### Economic analysis

Costs were calculated by combining the service use data with information on unit costs (Curtis, 2010). Lost employment was valued by combining lost work days with average earnings data. Costs at follow-up with and without lost employment were compared between the two groups. Due to expected skewness in the cost distribution, a bootstrapped regression model was used, with baseline costs controlled for.

Quality adjusted life years (QALYs) were computed from the EQ-5D by converting the five domain scores to previously established weights (Dolan *et al.* 1995) and using area under the curve methods. If the intervention results in lower costs and better outcomes then it is deemed ‘dominant’. If it, or the control, had both higher costs and better outcomes then an incremental cost-effectiveness ratio is calculated, defined as the difference in costs divided by the difference in QALYs. This shows the extra cost incurred to produce an extra QALY.

## Results

### Recruitment and representativeness of recruited participants

Participant flow through the trial is summarized in a consort diagram in Appendix 1. There were 9305 visits to the website, 1111 completed the screening and 637 were randomized, with 359 completing 6-week online assessments and 231 participants completing 12-week online assessments. The research portal was first piloted with organization A (communications sector), and then implemented incrementally there. It was promoted using internal communications and regular telephone conferences with key personnel over the duration of the study (2 years). Organization B (transport sector) joined the trial a few months later and promoted it through their intranet as well as referring individuals through the in-house counselling service, so offering it to employees who were deemed likely to benefit as well as to the wider workforce. After 1 year in the field, trial recruitment was lagging, so we recruited organization C (health sector) to the study in October 2010. This entailed making access available on their website, with minimal marketing. The recruitment numbers reflect the size of the employers and the nature of the workforces as well as the extent to which the study was actively promoted. At baseline assessment, organization A recruited 396 (62%), B recruited 100 (16%), and C recruited 141 participants (22%). Since the trial was open for different periods to each workplace, and Internet use is likely to vary between occupational settings, with high use in communications employees compared with transport employees, rates of recruitment cannot be compared meaningfully.

### Demographic comparisons

Table 2 shows the baseline demographic information according to trial arm. More males were randomized to control than MoodGYM; a sensitivity analysis therefore adjusted for gender. The occupational groups who participated in the study shown in Table 2 are not representative of their employers, nor of the personnel experiencing stress in each setting; they merely reflect the individuals who took up the trial opportunity.

**Table 2.** Characteristics of subjects according to study arm

	Control (*n* = 319)	MoodGYM (*n* = 318)
Gender		
Male	160 (50)	136 (43)
Female	152 (48)	176 (55)
Missing	7 (2)	6 (2)
Mean age, years (s.d.)	42.7 (9.6)	42.2 (9.6)
Marital status		
Single	82 (26)	67 (21)
Married/cohabiting	196 (61)	218 (69)
Widowed/separated	34 (11)	27 (8)
Missing	7 (2)	6 (2)
Organization^a^		
A	195 (61)	198 (62)
B	51 (16)	49 (15)
C	73 (23)	71 (22)
Occupation		
Manager or senior official	92 (29)	91 (29)
Professional	66 (21)	63 (20)
Associate professional or technical	33 (10)	32 (10)
Administrative and secretarial	34 (11)	52 (16)
Skilled trade	14 (4)	5 (2)
Personal services	0 (0)	2 (1)
Sales and customer service	60 (19)	61 (19)
Process, plant and machine operative	1 (0.3)	1 (0.3)
Other	19 (6)	10 (3)
Missing	0 (0)	1 (0.3)

s.d., Standard deviation.

Data are given as numbers (%) of subjects unless stated otherwise.

a A = communications sector; B = transport sector; C = health sector.

#### Clinical outcomes and missing data

We had data at one or more assessment points over the 12 weeks for 401 subjects (63%). Our primary outcome (WSAS scale) was completed by 636/637 subjects at baseline – the subject who failed to complete the baseline questionnaire was randomized and did complete the 6- and 12-week follow-ups as well as the MoodGYM modules. Completion was 359/637 (56%) at 6 weeks and 231/637 (36%) at 12 weeks.

As seen from these numbers, the completion rate of the initial 637 participants was low. We therefore compared the characteristics of completers with non-completers at both 6 and 12 weeks to see if there were any obvious differences between those who proceeded through the study and those who dropped out at 6 or 12 weeks. At 6 weeks the proportion completing follow-up was higher for the control arm but this difference was not statistically significant. The proportion of completers was higher in the transport sector workplace than in the health or communications workplaces; the finding was statistically significant but the differences were small. The only other finding of note was that the difference in ages was statistically significant; completers were marginally older. However, again, this difference was very small. Comparisons of the demographic information between completers and non-completers at 12 weeks revealed very similar patterns to the 6-week comparisons.

Baseline psychiatric scores were marginally lower in participants who completed the 6-week follow-up; these small differences were statistically significant. The same pattern was present at 12 weeks, apart from the WSAS score that showed that the non-completers had a marginally lower baseline WSAS score than the completers. However, none of these differences was statistically significant at 12 weeks.

#### Adherence to the experimental intervention

Each participant on MoodGYM should complete five modules, so to investigate adherence they were assigned a score from 0 to 4 to indicate the level of completion for each of these modules (0 = 0%, 1 = 25%, 2 = 50%, 3 = 75% and 4 = 100%). Thus each participant could score up to 20, and by inspecting the distribution of scores we found that the participants clustered into ‘high’ (9–20, *n* = 90), ‘medium’ (4–8, *n* = 117) and ‘low’ (0–3, *n* = 106) completion groups. We found no significant differences in the age, gender, marital status, median years of education or median units of alcohol consumed across high and low completion groups. It appears that those that completed the highest level of MoodGYM modules were marginally older but this difference was not statistically significant. There does not appear to be a significant difference in the median years of education or median units of alcohol consumed across the groups. Married individuals were over-represented in the high completion category compared with single/widowed/divorced – but this trend was not significant.

#### Primary analysis

**Fig. 1.**
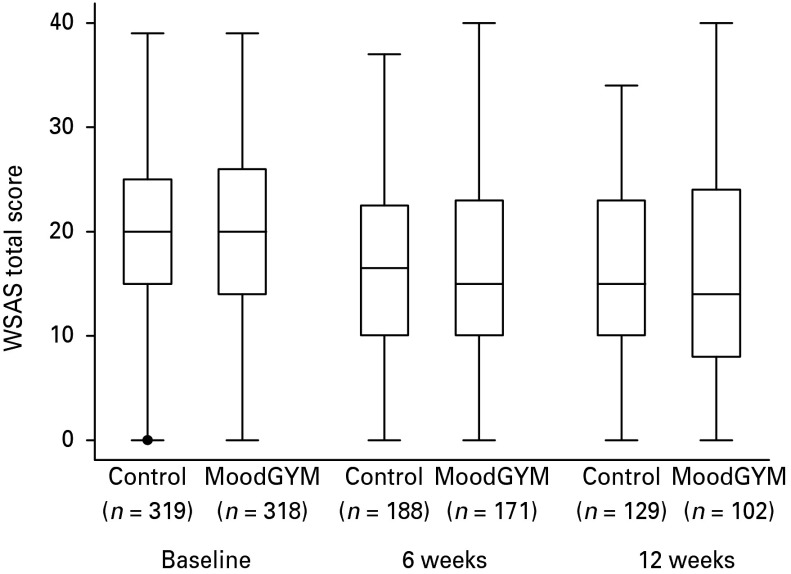
Box plots of Work and Social Adjustment Scale (WSAS) scores at each time point for the two trial arms. Data represent mean, interquartile range, minimum and maximum.

Table 3 shows the outcome scores by treatment arm at three time points. Means in both treatment arms decreased on each of the psychiatric measures. Fig. 1 illustrates the profile of the unadjusted means for the WSAS score. Adjusting for covariates (time, baseline score and organization) made little difference to the profile.\

**Table 3.** Comparison of intervention groups for primary and secondary outcome measures

Outcome	Control			MoodGYM			Treatment effect^a^^,^^b^ (95% CI); *p*
	*n*	Mean	(s.d.)	*n*	Mean	(s.d.)	
Primary							
WSAS							
Baseline	319	20.0	(7.7)	317	19.9	(8.0)	−0.470 (–1.837 to 0.897); 0.50
6 weeks	188	16.5	(8.6)	171	16.0	(9.1)	
12 weeks	129	15.9	(8.6)	102	15.0	(10.1)	
Secondary							
PHQ-9^c^							−0.429 (–1.454 to 0.595); 0.41
Baseline	318	14.6	(5.6)	311	14.6	(5.4)	
6 weeks	176	10.2	(6.0)	164	9.9	(6.1)	
12 weeks	122	10.3	(6.9)	97	9.3	(6.9)	
CORE10							
Baseline	318	18.3	(5.3)	316	18.4	(5.9)	−0.257 (–1.244 to 0.731); 0.61
6 weeks	187	15.3	(6.1)	171	15.3	(6.1)	
12 weeks	129	15.0	(6.9)	101	14.1	(7.3)	
GAD							−0.341 (–1.334 to 0.651); 0.50
Baseline	305	13.2	(5.0)	303	13.0	(5.4)	
6 weeks	181	10.2	(5.7)	166	9.5	(6.0)	
12 weeks	123	10.1	(6.5)	98	8.4	(6.4)	

s.d., Standard deviation; CI, confidence interval; WSAS, Work and Social Adjustment Scale; PHQ-9, Patient Health Questionnaire-9; CORE10, Clinical Outcomes in Routine Evaluation; GAD, generalized anxiety disorder.

a MoodGYM minus control, based on 6 and12 weeks combined, adjusted for time, baseline value of outcome and organization.

b Refers to the estimated mean difference, across both time points.

c Threshold for inclusion in the study was PHQ-9 score ⩾10.

In the random-effects model, which combines data from 6 and 12 weeks to give a combined effect size, there was no statistically significant difference in the average treatment effect on the WSAS score over the two time points. Also there was no evidence for an interaction between time point and intervention, and so no evidence of a differential effect over time (justifying the combination of the estimate of the effect over the two time points). This model adjusted for baseline WSAS score and organization. For the WSAS score the estimated treatment effect across follow-ups was –0.47 (95% CI –1.84 to 0.90, *p* = 0.5). There was no evidence to support a difference in the intervention effect according to employer (we tested for interaction). Nor was there evidence of a statistically significant treatment effect for any of the secondary outcomes.

We then compared the model for our primary analysis with two additional models, one to account for a possible gender imbalance at baseline, and another to control for any variables that were associated with missing follow-ups. Analysis showed that the following variables were associated with not completing follow-ups: age, organization and baseline psychiatric scores. These analyses did not give us any reason to alter our main finding that there was no evidence of a difference in effect between MoodGYM and control. There was no difference in primary outcome scores at 6 weeks for those who completed both follow-ups compared with those who only completed the 6-week follow-up (16.29 *v.* 16.28, respectively)

It was decided at the beginning of the study that the primary analysis would include both time points. However, at the end of the study when we could see that attrition was very large we re-ran the analysis exploring the differences between baseline and 6 weeks for exploratory purposes. The results of this did not change our conclusions.

### Economic results

There were no major differences in costs between the two groups at baseline. As shown in Table 4, in the 6 months prior to randomization at least two-thirds of subjects had GP contacts and one in five attended hospital as an out-patient. A similar proportion – about one-fifth of participants, used some kind of community-based provider. Most were in receipt of psychotropic medication: antidepressants and anxiolytics. During the 6-week follow-up period, there were no major differences in service use between the groups. Note that the baseline and follow-up costs relate to different time periods.

**Table 4.** Service use and costs at baseline and 6-week follow-up

Service	Baseline				Follow-up			
	MoodGYM intervention		Control		MoodGYM intervention		Control	
	Participants using, *n* (%)	Mean cost, all participants, UK£ (s.d.)	Participants using, *n* (%)	Mean cost, all participants, UK£ (s.d.)	Participants using, *n* (%)	Mean cost, all participants, UK£ (s.d.)	Participants using, *n* (%)	Mean cost, all participants, UK£ (s.d.)
Hospital services								
In-patient	20 (6)	101 (854)	27 (8)	345 (3733)	2 (1)	3 (46)	2 (1)	1 (22)
Out-patient	61 (19)	90 (748)	73 (23)	77 (315)	22 (7)	11 (73)	30 (9)	25 (118)
Community services								
GP	225 (71)	98 (187)	213 (67)	88 (130)	74 (23)	6 (17)	82 (26)	7 (18)
Psychiatrist	14 (4)	42 (484)	4 (1)	5 (52)	5 (2)	4 (38)	4 (1)	1 (10)
District nurse	44 (14)	4 (17)	46 (14)	3 (10)	11 (4)	0.12 (0.71)	19 (6)	1 (3)
Counsellor	47 (15)	33 (123)	52 (16)	36 (122)	20 (6)	2 (8)	23 (7)	2 (9)
Occupational health providers	35 (11)	3 (12)	44 (13.8)	6 (28)	7 (2)	0.5 (5)	18 (6)	3 (15)
Other providers	61 (19)	39 (254)	73 (23)	33 (143)	21 (7)	5 (44)	18 (6)	3 (15)
Medication	307 (97)		311 (98)		190 (60)		194 (61)	
Lost work								
Total absence	170 (54)	1240 (2996)	168 (53)	1137 (2643)	55 (17)	119 (440)	50 (16)	143 (906)
Due to illness	91 (29)	924 (2729)	86 (27)	818 (2526)	34 (11)	96 (417)	23 (7)	111 (898)
Total service cost	318	411 (1330)	319	592 (3898)		29 (110)		38 (125)
Total cost, including lost work		1651 (3531)		1730 (3531)		125 (451)		149 (908)

s.d., Standard deviation; GP, general practitioner.

At baseline, around half the sample had taken time off work due to sickness, on average about 20 days over 6 months. Respondents stated that most of this time (52%) was taken off due to their mental health, which affected 28% of employees. Participants who were absent for this reason took longer on average to return to work – about 28 days. About 16% of participants took some time off sick during the study period – on average 7 days. Most of this sick leave (9% of employees, 56% of those off work) was attributed to mental health difficulties, and again the average length of absence was longer than for other health reasons: 11 days.

The cost of lost employment was higher for the control group at follow-up (£111 *v.* £96, a difference of 2 days on average over all participants during the 5-week follow-up period). However, this did not attain statistical significance (*t* test of independent means: *p* = 0.759, 95% CI –126 to 92). A similar pattern was seen in the total costs of absence from work, where control participants had higher costs (£143 *v.* £119) but this was not significant (*p* = 0.644, 95% CI –137 to 84).

Over the first 6 weeks of the follow-up there was virtually no difference in QALYs gained (0.082 MoodGYM, 0.083 control). Over the 12 weeks the gains were 0.170 and 0.167, respectively. In terms of point estimates, MoodGYM resulted in slightly lower costs but a slightly lower QALY gain. However, the uncertainty around the estimates is suggestive of no apparent difference in cost-effectiveness between the groups.

## Discussion

Most subjects participating in both arms of the trial improved over time and returned to work. Compared with the control group outcomes, MoodGYM was not associated with greater improvement in health or quality of life over the follow-up period. Both arms received regular telephone calls to collect data and divert participants at risk; this constant should be borne in mind when interpreting the results. It is possible that merely receiving a weekly telephone call concerning one's service use and symptoms had beneficial effects, irrespective of whether participants used the online resources, although Christensen *et al.* (2004) found a difference between telephone-only controls and a self-selected intervention group. The lack in the current study of a control group that received no direct human contact of any kind means that this cannot be ruled out. An alternative explanation might be a placebo effect from Internet use; a further control group not exposed to any form of Internet use would be required to test this, but that is not consistent with the pragmatic design of this trial.

It is difficult to distinguish the immediate cause of any given period of sickness absence. In this study we simply asked the participants to tell us if their time off was due to their mental health, and indeed this was the attribution of most of the time off which was reported, both in the 6 months preceding the study and during the 5-week intervention period. Although not statistically significant, and with substantial variation about the mean, average sick leave absence of two additional days over 5 weeks for the control group extrapolates to an additional 4 weeks' absence per annum. While this could well be a chance finding, it may be taken as grounds for further investigation.

A notable aspect of this study is its approach to recruitment, screening and participation online, whose potential applications in clinical research have yet to be fully realised. This proved challenging and instructive. The experience gained in this trial has helped to build capacity for future studies and advance knowledge around benefits and obstacles of trial implementation through the Internet. Besides cost efficiency, online data collection offers the ability to involve people from anywhere in the world, rigorously consistent delivery of designated interventions, a certainty that consent is genuine and on-going, and minimal researcher bias, since the research team had no contact with the participants.

### Limitations

Despite telephone calls and email prompts to complete assessment tools, the retention rate from this study (56% at 6 weeks, 36% at 12 weeks) was low by comparison with studies where there is face-to-face contact between the participant and the research team. This and the relatively short follow-up period are the most serious limitations of this study. Consequently we do not know whether the recovery rates for participants in the MoodGYM and control arms continued in parallel over time or not.

A key disadvantage seems to be that there are limited ways to maximize retention of participants in an online study, since engagement relies on individual initiative and commitment. The delivery of the trial in a clinical setting or via participants' physicians seems to be consistently more effective in terms of their retention. Pittaway *et al.* (2009), whose participants were referred by GPs but whose intervention took place outside the surgery settings, had a 50% completion rate. In a general practice-based study of Beating the Blues, Proudfoot *et al.* (2004) retained 74% of their sample between baseline and post-intervention assessment, and in a smaller primary care sample (*n* = 48) Grime (2004) retained 90% at follow-up. However, our study was relatively ‘arm's length’ and relied on the motivation of participants without the reinforcement of their clinicians.

Nonetheless, our sample showed greater commitment than the community-based participants in a MoodGYM trial by Christensen *et al.* (2006), which found that only 30% of those individuals who complied with the baseline assessment completed even one module of the programme. The latter was a self-selected sample, a diagnosis of depression was not made and there was no email or telephone contact with participants. However, the same team undertook a three-way trial of MoodGYM, BluePages (a psycho-educational site) and a treatment-as-usual control (Christensen *et al.* 2004) – and achieved a completion rate of 81%.

We found that more participants were lost to follow-up from the MoodGYM arm than from the control arm, although this was not significant. Christensen *et al.* (2004) also found that MoodGYM subjects were more likely to drop out than participants using BluePages (25% *v.* 15%). Such a trend could be due to the more demanding requirements of the interactive CBT aspects of MoodGYM with its exercises and quizzes to complete. This implies a need to screen for user suitability for CBT, with the provision of alternative resources for those for whom it is not suitable.

There may also be scope to influence potential participants' motivation to comply with the requirements of cCBT, perhaps through education or by offering incentives. Tailoring the pace of delivery or the number of exercises to complete each week to individual preferences may also help with retention. Computerized intervention packages could probably increase their appeal by learning from developments in online marketing.

## Conclusion

Although the hypothesis that MoodGYM would have superior outcomes was not proven, given the large numbers of employees affected by common mental health problems, employers stand to gain from further research in this area, while health providers within employing organizations and beyond may find that judicious use of online self-help can reduce demand for face-to-face therapy and cut costs.

Retention is fundamental to the successful take-up of such interventions, even before their effectiveness can be rigorously tested. More work is needed to streamline the delivery of online resources, and data collected through this study can be used to inform such developments.

The scope of online interventions is largely restricted to common mental disorders broadly defined. Different packages may need to be developed for particular difficulties, for example bullying at work or bereavement, both of which can manifest as depression with or without anxiety.

No adverse outcomes were found from the trial, which extended over a large number of participants and nearly 2 years in time. This may be taken to add to the evidence that online self-help is safe. It seems likely that different combinations of self-help and professional input, in person or online, will suit different people. What works best for whom is not clear, so it is reasonable to encourage people to discover for themselves whether cCBT can aid their recovery. This study functions as a demonstration of the strengths and weaknesses of Internet-based research on psychological well-being, one that can serve both as a cautionary tale and as a platform for new research.

## Figures and Tables

**Figure:**
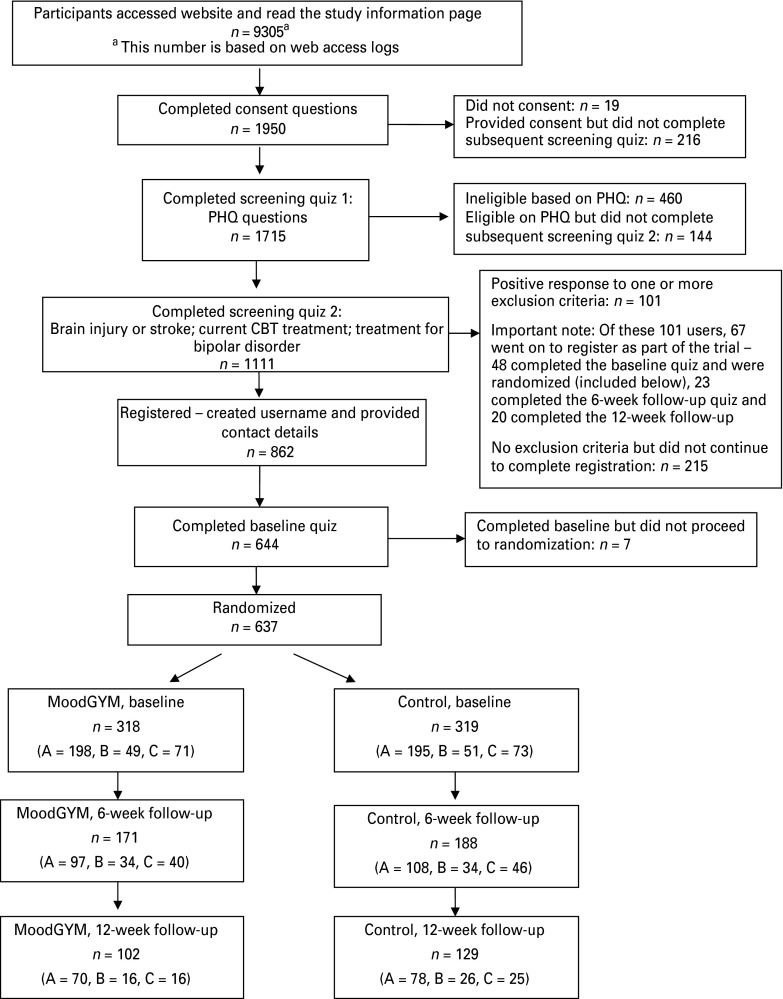
PHQ-9, Patient Health Questionnaire-9; CBT, cognitive behavioural therapy; A, communications sector; B, transport sector; C, health sector.
